# Investigating years of life lost in Belgium, 2004–2019: A comprehensive analysis using a probabilistic redistribution approach

**DOI:** 10.1186/s13690-023-01163-7

**Published:** 2023-08-25

**Authors:** Brecht Devleesschauwer, Aline Scohy, Robby De Pauw, Vanessa Gorasso, Anne Kongs, Elias Neirynck, Peter Verduyckt, Grant M. A. Wyper, Laura Van den Borre

**Affiliations:** 1 Service Health Information, Department of Epidemiology and Public Health, Sciensano, Rue Juliette Wytsman 14, Brussels, 1050 Belgium; 2 Department of Translational Physiology, Infectiology and Public Health, Ghent University, Merelbeke, Belgium; 3 Department of Rehabilitation Sciences, Ghent University, Ghent, Belgium; 4 Department of Public Health and Primary Care, Ghent University, Ghent, Belgium; 5 Department of Care, Flemish Public Administration, Brussels, Belgium; 6 Statistics Belgium, Brussels, Belgium; 7 Brussels-Capital Health and Social Observatory, Brussels, Belgium; 8 Place and Wellbeing Directorate, Public Health Scotland, Glasgow, UK; 9 School of Health & Wellbeing, University of Glasgow, Glasgow, UK; 10 Interface Demography, Department of Sociology, Vrije Universiteit Brussel, Brussels, Belgium

**Keywords:** Cause of death, Mortality, Years of life lost, Burden of disease, Redistribution, Garbage code, Ill-defined death, Belgium

## Abstract

**Introduction:**

Information on years of life lost (YLL) due to premature mortality is instrumental to assess the fatal impact of disease and necessary for the calculation of Belgian disability-adjusted life years (DALYs). This study presents a novel method to reallocate causes of death data.

**Materials and methods:**

Causes of death data are provided by Statistics Belgium (Statbel). First, the specific ICD-10 codes that define the underlying cause of death are mapped to the GBD cause list. Second, ill-defined deaths (IDDs) are redistributed to specific ICD-10 codes. A four-step probabilistic redistribution was developed to fit the Belgian context: redistribution using predefined ICD codes, redistribution using multiple causes of death data, internal redistribution, and redistribution to all causes. Finally, we used the GBD 2019 reference life table to calculate Standard Expected Years of Life Lost (SEYLL).

**Results:**

In Belgium, between 2004 and 2019, IDDs increased from 31 to 34% of all deaths. The majority was redistributed using predefined ICD codes (14–15%), followed by the redistribution using multiple causes of death data (10–12%). The total number of SEYLL decreased from 1.83 to 1.73 million per year. In 2019, the top cause of SEYLL was lung cancer with a share of 8.5%, followed by ischemic heart disease (8.1%) and Alzheimer’s disease and other dementias (5.7%). All results are available in an online tool https://burden.sciensano.be/shiny/mortality2019/.

**Conclusion:**

The redistribution process assigned a specific cause of death to all deaths in Belgium, making it possible to investigate the full mortality burden for the first time. A large number of estimates were produced to estimate SEYLL by age, sex, and region for a large number of causes of death and every year between 2004 and 2019. These estimates are important stepping stones for future investigations on Disability-Adjusted Life Years (DALYs) in Belgium.

**Supplementary Information:**

The online version contains supplementary material available at 10.1186/s13690-023-01163-7.

**Table:** 

**Text box 1. Contributions to the literature**
• Ill-defined deaths (34% of all deaths in Belgium) pose challenges in estimating the burden of disease, particularly in an aging population.
• A four-step method was developed to redistribute ill-defined deaths, providing more accurate estimates of causes such as ischemic heart disease, cerebrovascular disease, and lung cancer.
• Mortality rates decreased over time, except for Alzheimer’s disease and other dementias, emphasizing the need for targeted public health strategies. Variations in mortality patterns were observed by sex and region.

## Introduction

Burden of Disease (BoD) studies are increasingly used to assess the population health impact of different health conditions. One of the key metrics for quantifying BoD is the Disability-Adjusted Life Years (DALYs), which represents the “health gap” between the observed population health state and a hypothetical one in which everybody reaches old age free of disease or injury [1]. Specifically, DALYs summarize the years of life lost due to premature mortality (YLL) and the years of life spent with disability (YLD).

The presence of ill-defined deaths (IDD) in death registration systems is a major issue for the calculation of YLL, and thus DALYs. Generally, official mortality statistics are based on the underlying cause of death, which is the disease or injury that started the chain of events leading to death [2]. However, not all coded underlying causes of death are suitable to estimate the BoD [3]. Some codes are too unspecific (e.g., ICD-10 Y34 “Unspecified event, undetermined intent”), while other codes represent inaccurate causal sequences (e.g., ICD-10 I46 “Cardiac arrest” as the underlying cause of death).

Ill-defined causes of death need to be dealt with to depict an accurate assessment of national disease burdens. There are large variations in the quality of cause-specific mortality data between countries. An international comparison found that 7% (Finland) to 66% (Egypt) of the national cause-specific mortality data was not accurate enough to estimate BoD [4]. One of the key challenges is to make optimal use of the available data. To estimate the BoD accurately, the IDD (also called ‘garbage codes’) need to be redistributed to valid categories of causes of death (i.e., target codes) [5].

The current literature presents a number of methods to redistribute garbage codes. One methodological approach uses multiple causes of death data [6–9]. In addition to the underlying cause of death, death certificates often contain the sequence of events or diseases that resulted in death, as well as other causes contributing to death. This additional information on diseases that accompany or (in)directly cause the death can be used to verify the registered underlying cause of death or complement the information in the coded underlying cause of death. One of the most well-known methods was developed by Naghavi and colleagues within the framework of the Global Burden of Disease (GBD) study integrating fractional reassignment of death due to multiple causes, proportional reassignment, regression models, and redistribution based on fixed proportions [10]. The algorithm was last revised in GBD 2013 with some minor updates in GBD 2019 and GBD 2020, and can be used to derive country-specific estimates [5, 11]. However, the methodology used is complex and builds on several assumptions which do not necessarily reflect the national context [12]. General redistribution techniques may generate biased estimates due to differential patterns in IDDs observed between and within countries [4, 13]. It appears that GBD estimates are useful to compare population health between countries but are perhaps less appropriate for comparisons within countries. However, many national decision-making processes rely on those internal comparisons over time and between groups. To this end, there is a growing body of literature on national BoD assessments.[e.g., 9, 14–16] In Belgium, the national institute for health, Sciensano, initiated the Belgian national burden of disease study (BeBOD) in 2016, to generate DALY estimates rooted in local data and evidence. This article presents the four-step method used in BeBOD to redistribute IDDs making use of multiple causes of death data, and provides a quantification of the fatal BoD in Belgium for the period 2004–2019.

## Materials and methods

### Causes of death data

Death certificates in Belgium are based on the World Health Organization (WHO) International Form of Medical Certificate of Cause of Death. The certifying physician describes the chain of events or diseases that finally caused death and the associated health problems or events that played a role in the process. The completed death certificates are collected by the municipal offices, and sent to the regional health authorities, i.e., the Flemish Agency for Care and Health (for deaths occurring in the Flemish Region), the Brussels-Capital Health and Social Observatory (for deaths occurring in the Brussels Capital Region), and the Walloon Agency for a Quality Life (for deaths occurring in the Walloon Region). The Flemish Agency for Care and Health and Walloon Agency for a Quality Life encode the information listed on the death certificates into ICD-10 codes, defining the underlying cause of death according to the ICD-10 rules of the WHO. The resulting datasets are compiled and coupled to the National Register by Statistics Belgium (Statbel), the national institute of statistics, which is responsible for managing the national causes of death database. Statbel checks the data quality of socio-demographic variables and the underlying cause of death according to Eurostat guidelines. Sciensano receives the national dataset for further analysis.

The process of compiling the national causes of death data currently takes around 2 years. Delays occur in the various stages of the complex data transfer starting with the administration related to the funeral, the death certificate transferring to the municipalities, and then later to the regions. Non-natural deaths are further investigated by the prosecutor offices, which may add to the delay. Moreover, rigorous verifications are performed by the regional offices who may contact the certifying doctors in order to identify the most correct and specific underlying cause of death. We use nationally representative causes of death data from 2000 to 2019 for this analysis. Completeness of demographic data in the national causes of death database is very high with information on age, sex, and place of residence. Across the study period, only 8 deaths were identified with missing information on age, and 5 deaths with missing information on sex. These cases were excluded from further analyses.

### Cause mapping

As a first step in the analysis process, we mapped the ICD-10 codes of the underlying cause of death (UC) to the GBD cause list [5]. Since a detailed map from the GBD is not available, we adopted the map used by the Scottish Public Health Observatory [17], with some modifications (Supplementary File 1). Additionally, for drug-related deaths, we followed the definitions from the European Monitoring Centre for Drugs and Drug Addiction for combinations of ICD-10 × 41, X44, X61, X64 or Y11 with T43.6, and X42, X62, or Y12 with T40 (mapped to ICD-10 F19) [18]. In line with GBD, stillbirths are excluded from the analyses. The final cause map included 130 causes of death (level 3), grouped into 20 disease groups (level 2), each classified into one of the three GBD clusters (level 1), i.e., non-communicable diseases (NCD), communicable, maternal, neonatal and nutritional diseases, and injuries. Various level 3 causes are further subdivided into two or more level 4 causes. We performed our mapping process at level 4, but present results at level 3. In the mapping process, several IDD ICD-10 codes could not be matched with a specific level 4 GBD cause. The next section describes our approach to redistributing these so called “ill-defined deaths” (IDDs) to specific GBD causes.

### Redistribution of ill-defined deaths

To redistribute the IDDs to specific GBD causes, we developed a probabilistic approach making use of the multiple causes of death data available in Belgium. Our approach consists of four different steps, with at each step an update of the distribution of the target codes. These updated target distributions were used at each step and for each group within a given step. This is to respect the sequence of redistributions and the build-up of evidence along the redistribution process.

In the first step, selected UCs of IDDs are proportionally redistributed in function of predefined ICD-10 target codes (“ICD-based redistribution”, Supplementary File 1). The predefined ICD-10 target codes have been adapted by Sciensano based on the methodology applied in Scotland [17]. For example, malignant neoplasm of the uterus, part unspecified (ICD-10 C55) is redistributed to two groups of target codes – malignant neoplasm of cervix uteri (ICD-10 C53) and of corpus uteri (ICD-10 C54) – pro rata to the occurrence of both diseases as underlying causes of death. To incorporate recent trends and reduce random variation, the target distributions are defined based on the deaths that occurred in the past five years (i.e., the targets for IDDs in year $y$ are based on specific deaths occurring in year $y-4$ to $y$). This also implies that the first estimates are available for the year 2004, i.e., the first year for which a five-year period of causes of death data are available. To add to precision, the target distributions are stratified by age group (0–4, 5–14, 15–44, 45–64, 65–84, 85+) and sex. If there are very small numbers of deaths in a given combination, a target distribution based on sex only was used. If there are no observed deaths in the target group, then the IDDs are proportionally redistributed to all causes. We also exclude sex-specific diseases or health related problems from the target distributions of the opposite sex, e.g., we ensure that deaths in females could not be redistributed to prostate cancer.

The first step in the redistribution process is only applicable to IDDs with codes providing clear information on the possible underlying cause of death, and thus allow defining target codes a priori. In the second step, we rely on the Belgian multiple causes of death data to define targets and redistribute selected remaining IDDs that are coded an intrinsically uninformative UC code (“package redistribution”). For example, the assigned underlying cause of death is “Unspecified kidney failure” (ICD-10 N19). This code cannot be assigned to a specific GBD cause (it is an IDD) and there are no predefined target codes available (step 1). For these cases, we defined packages, i.e., sets of IDDs that are similar and considered to have a similar redistribution target (Supplementary File 1). In this example, the package created is called “Acute kidney failure” and is made of the following ill-defined ICD-10 codes: N19, N17.0, and N17.9. For each package, the target distribution is defined as the deaths, occurring in the past five years, that have one of the package ICD-10 codes as immediate, intermediate, or associated cause of death, and that have a specific or redistributed cause as underlying cause of death. As before, the redistribution is performed proportionally to the observed target codes, stratified by age group and sex. Because the target is defined in function of the multiple causes of death data of the preceding 5-year period, the targets are not fixed and may differ for the different years of the time series.

The remaining IDDs are those that are assigned an uninformative UC code. These include some codes of ICD-chapter R, “Symptoms, signs and abnormal clinical and laboratory findings, not elsewhere classified”. To maximize the use of the available multiple causes of death data, we apply a third step where we perform an internal redistribution of IDDs (“internal redistribution”). In this approach, remaining IDDs are randomly assigned to a specific GBD cause that is mentioned on the death certificate of the deceased individual.

In the fourth and final step, all remaining IDDs are proportionally redistributed over all specific causes having occurred in the preceding five years, stratified by age group and sex as described above (“all-cause redistribution”).

GBD causes (level 3) with less than five occurrences in the five-year reference period (i.e., less than one per year on average), are excluded as possible targets. These rare causes (e.g., rabies (A82)) are typically very specific diseases, with a specific diagnosis, and would therefore be rarely missed as underlying cause of death.

To capture the uncertainty that results from the probabilistic redistributions, the redistribution process was performed in a probabilistic way, using 100 iterations. Each iteration is a complete run of the four-step process and results in a completely imputed cause of death dataset. For each of the summary statistics (as described below), 100 random estimates are thus generated. We present means and 95% uncertainty intervals of these 100 random estimates, the latter defined as the 2.5th and 97.5th percentile of the random estimates.

### Quantification of the fatal burden of disease in Belgium

In addition to calculating the number of deaths, the fatal BoD in Belgium was also assessed using the Standard Expected Years of Life Lost (SEYLL), which is a well-established measure for the burden of premature mortality [19]. SEYLL can be defined as the total number of years that persons in a population would have lived if they had not died prematurely due to a disease or injury, against an aspirational life expectance based on the lowest age-specific mortality rates observed across time and geographical locations. Deaths at each specific age can be weighted by the expected number of years lost at each age. Previous assessments of Belgian premature mortality used fixed cut-off ages meaning that deaths at ages above the cut-off year did not contribute to the quantification of the burden of premature mortality (thus calculating Potential Years of Life Lost) [e.g., 20, 21]. We used the GBD 2019 reference life table for calculating the years of life lost for each age group following the recommendations made in previous studies [22–24]. We used linear interpolation to define SEYLL for specific ages. The GBD reference life table ends at age 95. We completed the life table by assuming a SEYLL of 0 for death at the age of 122, the highest observed lifespan worldwide to date [25].

The resulting number of deaths and SEYLL are presented as absolute numbers, crude rates (per 100,000 inhabitants), and age-standardized rates based on five-year age groups using both the 2013 European standard population [26] and the Belgian standard population, defined as the official population residing in Belgium on 1 January 2019 [27]. In this article, the focus will be on presenting the age-standardized rates by sex and region based on the European standard population. To gain further insights into the redistribution procedure, results further include the proportion of IDD out of all deaths by cause.

### Availability of code and estimates

All analyses were performed in R 4.1.0 [21]. The R scripts and inputs for the mapping and redistributions are available via GitHub (https://github.com/sciensanogit/BeBOD and https://github.com/sciensanogit/SEYLL2019). All estimates presented in this manuscript can be explored via https://burden.sciensano.be/shiny/mortality2019. Subsequent updates to the estimates will be available via https://burden.sciensano.be/shiny/mortality.

## Results

### Redistribution of ill-defined deaths

The percentage of IDDs increased from 31 to 34% between 2004 and 2019. Individuals with an ill-defined underlying cause of death were on average older than individuals with a specific underlying cause of death (Mean age: 80 vs. 75 years). IDDs were more common in female (36%) than in male (28%). The percentage of IDDs was slightly higher in the Brussels Capital Region (33%) than in the Flemish (32%) and Walloon Region (31%).

**Fig. 1:**
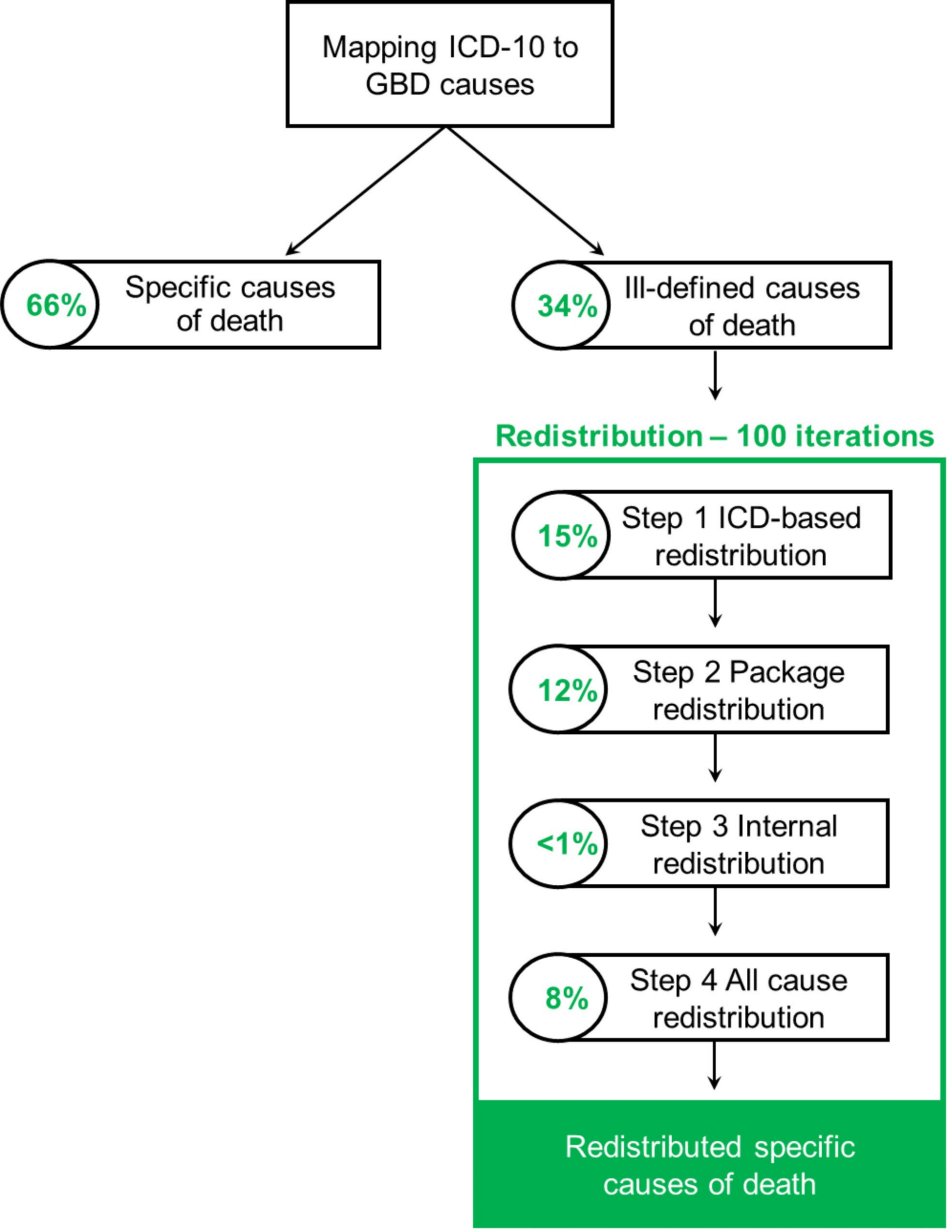
Visualisation of the redistribution method with the percentage of redistributed deaths per step in 2019

Among the 31–34% of IDDs, the majority of IDDs were redistributed using predefined ICD codes in step 1 (14–15% of total deaths), followed by the package redistribution in step 2 (10–12%). Only a minority of IDDs were redistributed using the internal redistribution approach in step 3 (< 1%). The remaining IDDs were redistributed to all causes in step 4 (5–8%). Figure 1 provides a visualisation of the redistribution procedure for the data in 2019.

The most common ill-defined UCs were I50.9 (Heart failure, unspecified), followed by J18.9 (Pneumonia, unspecified), and R99 (Ill-defined and unknown cause of mortality). These ill-defined UCs are among the main causes of death in Belgium. Heart failure was the fifth leading cause of death in 2019 despite some discussion on whether this code can be used for UC [2].

**Fig. 2:**
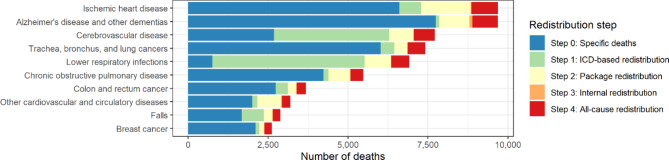
Estimated number of deaths in 2019 by level 3 GBD cause, for the top 10 causes of deaths, with breakdown by the contribution of the different steps in the redistribution process

At the end of the redistribution process, the causes with the largest number of redistributed IDDs were lower respiratory infections, cerebrovascular diseases, and ischemic heart disease (Fig. 2). Lower respiratory infections had the largest share of redistributed deaths using pre-defined target codes (step 1), mainly due to J18.9 (Pneumonia, unspecified organism) followed by cerebrovascular disease, mainly due to I64 (Stroke, not specified as haemorrhage or infarction). Ischemic heart disease had the largest share of package redistribution (step 2). Alzheimer’s disease and other dementias, and adverse effects of medical treatment had the largest share of internal redistribution (step 3).

### Belgian fatal burden of disease, 2004–2019

The redistribution method was used to quantify the national fatal burden of disease in Belgium for the period 2004–2019. In the first section, mortality patterns are gauged to examine which were the most relevant diseases or injuries in Belgium and how the impact of these causes of death changed over time. The second section focuses on premature mortality, as measured by the SEYLL.

### Deaths

**Fig. 3:**
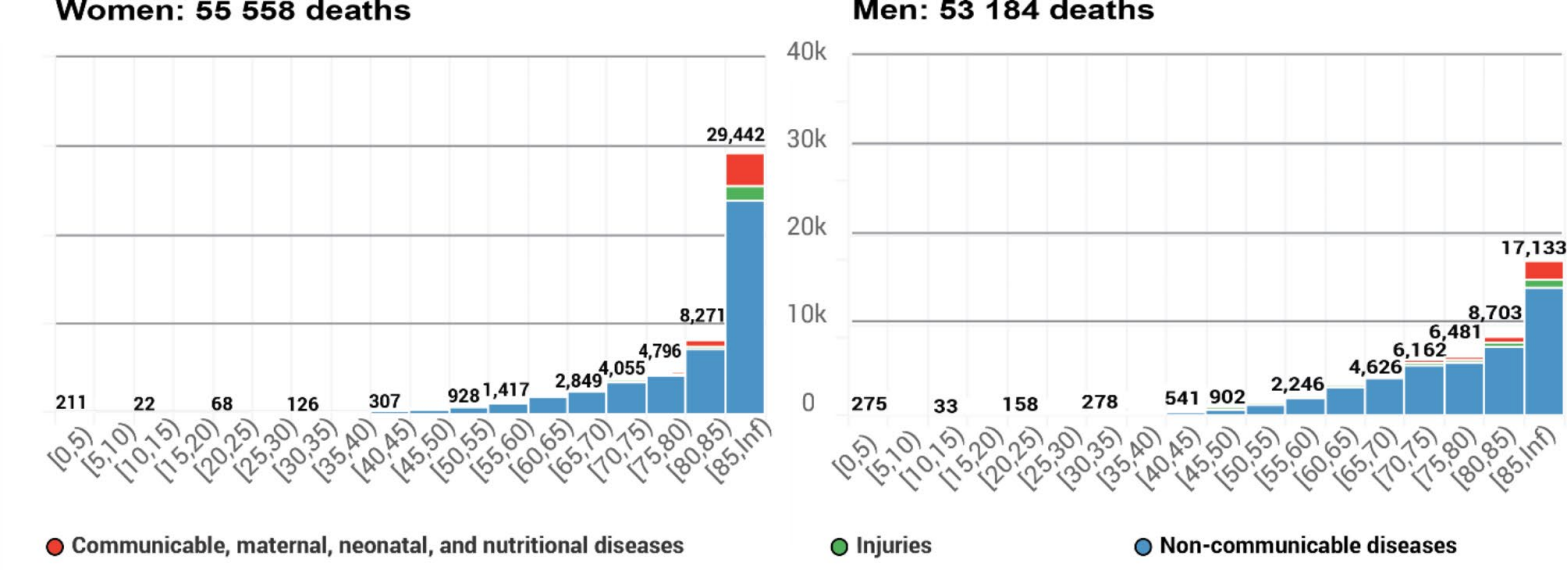
Absolute number of deaths in 2019, by sex and age group

The total number of deaths among Belgian residents increased from 101,250 to 2004 (972 per 100,000) to 108,742 in 2019 (949 per 100,000). Whereas male and female deaths were equally distributed in 2004, the mortality data for 2019 showed a slight relative increase with 51% female deaths versus 49% male deaths. Most deaths occurred at advanced ages, with 77% of female deaths and 61% of male deaths occurring at the age of 75 or above in 2019 (Fig. 3).

**Fig. 4:**
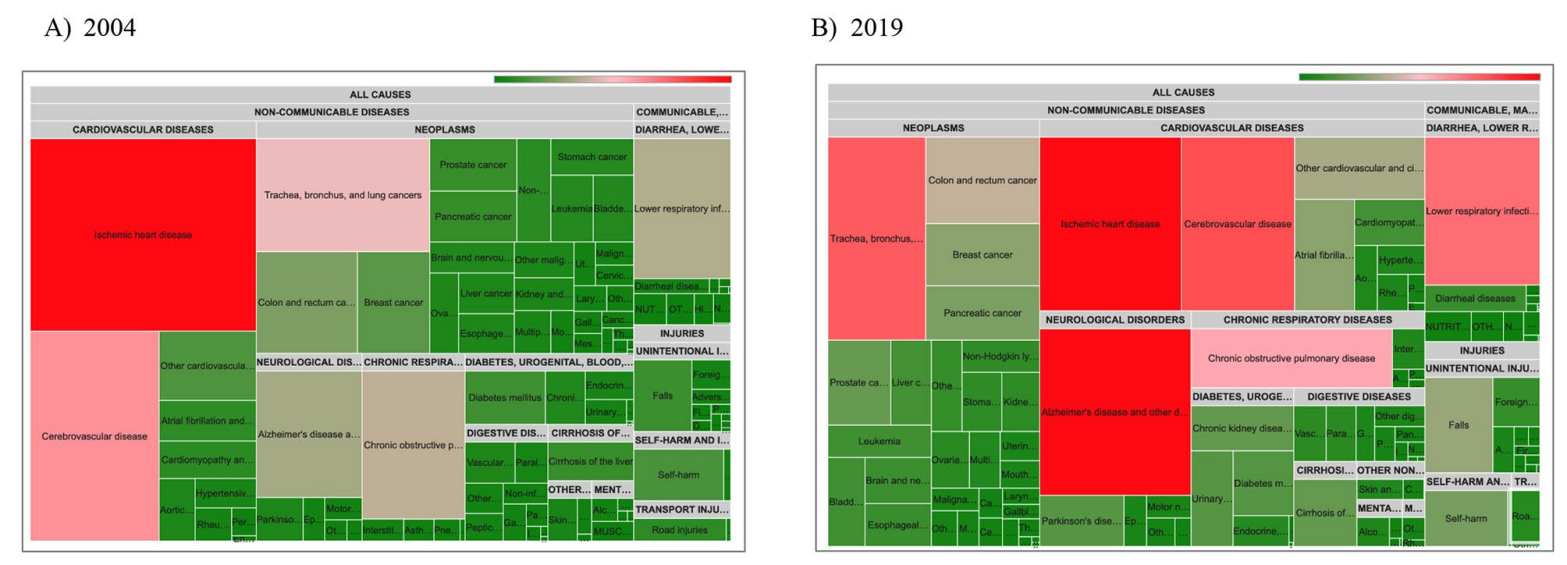
Treemaps visualizing the number of deaths by GBD cause for the total Belgian population in (**A**) 2004 and (**B**) 2019

When considering the major disease and injury groups (Fig. 4), non-communicable diseases were the dominant cause of mortality, although their share decreased from 86% to 2004 to 83% in 2019. The share of communicable diseases increased from 7 to 9%, and that of injuries from 7 to 8%. A closer inspection of the subcategories (level 2) showed that in 2004, cardiovascular diseases were the dominant cause of death group (32%), followed by neoplasms (29%); in 2019, neoplasms had become the dominant group (30%), with cardiovascular diseases reduced to the second place (24%). Neurological diseases ranked third throughout the study period, increasing from a share of 6.7% in 2004 to 12% in 2019. At the specific disease and injury level (level 3), ischemic heart disease was the dominant cause of death across the study period, but with a share that decreased from 15 to 9%. In 2004, cerebrovascular diseases (9%) and lung cancer (7%) completed the leading three causes, while in 2019 this was Alzheimer’s disease and other dementias (9%), and cerebrovascular diseases (7%).

Taking differences in population size and age structure into account, the age-standardized mortality rates (ASMRs) showed a decrease from 1,167 to 100,000 inhabitants in 2004 to 903 per 100,000 in 2019. Both male and female ASMRs decreased over time, albeit at a slower pace among females. Male ASMRs declined by 27% from 1,485 to 1,088 per 100,000 whereas female ASMRs were reduced by 20% from 951 to 759 per 100,000. However, male mortality remains 43% higher than female mortality in 2019.

Although age-standardized mortality rates decreased in all regions, rates were consistently higher in the Walloon Region, followed by the Brussels Capital Region, and lowest in the Flemish Region. The mortality gap between the Walloon Region and the Flemish Region increased from 10% to 2004 to 20% in 2019.

Table 1 presents the ASMRs for the ten leading GBD causes of death (level 3) in 2019. The highest cause-specific ASMRs were estimated for ischemic heart disease (80 per 100,000 inhabitants), Alzheimer’s disease and other dementias (77 per 100,000), lung cancer (65 per 100,000), cerebrovascular diseases (63 per 100,000), and lower respiratory infections (55 per 100,000). Within the leading five causes, only Alzheimer’s disease and other dementias showed increasing ASMRs between 2004 and 2019 (+ 21%). Specifically for female, a significant increase in lung cancer AMSRs was observed (+ 38%).

The above-mentioned diseases also made up the leading five causes of death for the Flemish, Brussels, and Walloon regions with some differences in the order of the ranking by region.

When considering differences in the importance of causes of death by age group, results showed that lung cancer was the leading cause of death for the age groups between 55 and 75. For 80-year-olds and older, Alzheimer’s disease and other dementias, and ischemic heart diseases were the main cause of death for elderly female and male, respectively.

**Table 1:** Leading 10 GBD causes of death (level 3) by sex in function of age-standardized mortality rates (ASMR) in 2019 and change with the ASMR in 2004 in percent. Reference: 2013 European Standard Population

	Both sexes				Male					Female			
**Rank**	*Cause*	*ASMR* *2019*	*% change with 2004*		*Cause*	*ASMR* *2019*	*% change with 2004*				*Cause*	*ASMR* *2019*	*% change with 2004*
1	Ischemic heart disease	80.3	-56%		Ischemic heart disease	112.8	-55%				Alzheimer’s disease and other dementias	79.6	22%
2	Alzheimer’s disease and other dementias	77.0	21%		Trachea, bronchus, and lung cancers	97.5	-34%				Cerebrovascular disease	59.2	-47%
3	Trachea, bronchus, and lung cancers	64.8	-17%		Alzheimer’s disease and other dementias	70.7	23%				Ischemic heart disease	56.0	-59%
4	Cerebrovascular disease	63.0	-46%		Lower respiratory infections	67.0	-26%				Lower respiratory infections	47.6	-15%
5	Lower respiratory infections	55.5	-19%		Cerebrovascular disease	67.0	-44%				Trachea, bronchus, and lung cancers	39.9	38%
6	Chronic obstructive pulmonary disease	46.7	-30%		Chronic obstructive pulmonary disease	66.5	-44%				Breast cancer	39.2	-25%
7	Colon and rectum cancer	31.1	-28%		Prostate cancer	41.6	-27%				Chronic obstructive pulmonary disease	33.9	-12%
8	Other cardiovascular and circulatory diseases	25.5	-13%		Colon and rectum cancer	37.6	-29%				Colon and rectum cancer	26.1	-28%
9	Falls	23.6	7%		Self-harm	29.2	-12%				Other cardiovascular and circulatory diseases	25.0	-13%
10	Breast cancer	22.3	-27%		Falls	26.6	11%				Atrial fibrillation and flutter	22.0	19%

### Standard expected years of life lost

The total number of SEYLL decreased over the study period from 1.83 to 1.73 million per year. Non communicable diseases were the dominant cause of SEYLL (81–82%), followed by injuries (11–12%) and communicable diseases (6–8%). Neoplasms were almost constantly the leading cause of SEYLL group with a share ranging between 33 and 36%. Cardiovascular diseases were the second leading group, with a share that decreased from 26 to 20%. The leading three causes was completed by neurological diseases, that has become more burdensome over time with a mortality share of 5% in 2004 and 9% in 2019. At the specific disease and injury level (level 3), ischemic heart disease ranked first in 2004 with a share of 13%, followed by lung cancer (9%) and cerebrovascular disease (7%). In 2019, lung cancer ranked first (9%); followed closely by ischemic heart disease (8%), and Alzheimer’s disease and other dementias (6%).

Age-standardized SEYLL rates (ASYRs) decreased from 19,555 to 100,000 inhabitants to 14,870 per 100,000 between 2004 and 2019. The patterns in ASYRs by sex and region are similar to those observed for mortality. A sharp decline in ASYRs was found among males (-28%) and females (-20%) over time. Despite a clear convergence of male and female trends, the 2019 ASYRs for males were still 57% higher compared to those for females. Regional ASYRs dropped over time with consistently higher estimates in the Walloon Region, followed by the Brussels Capital Region, and the Flemish Region. Compared with the Flemish Region, the ASYRs for the Walloon Region were 16% and 25% higher in 2004 and 2019, respectively.

**Fig. 5:**
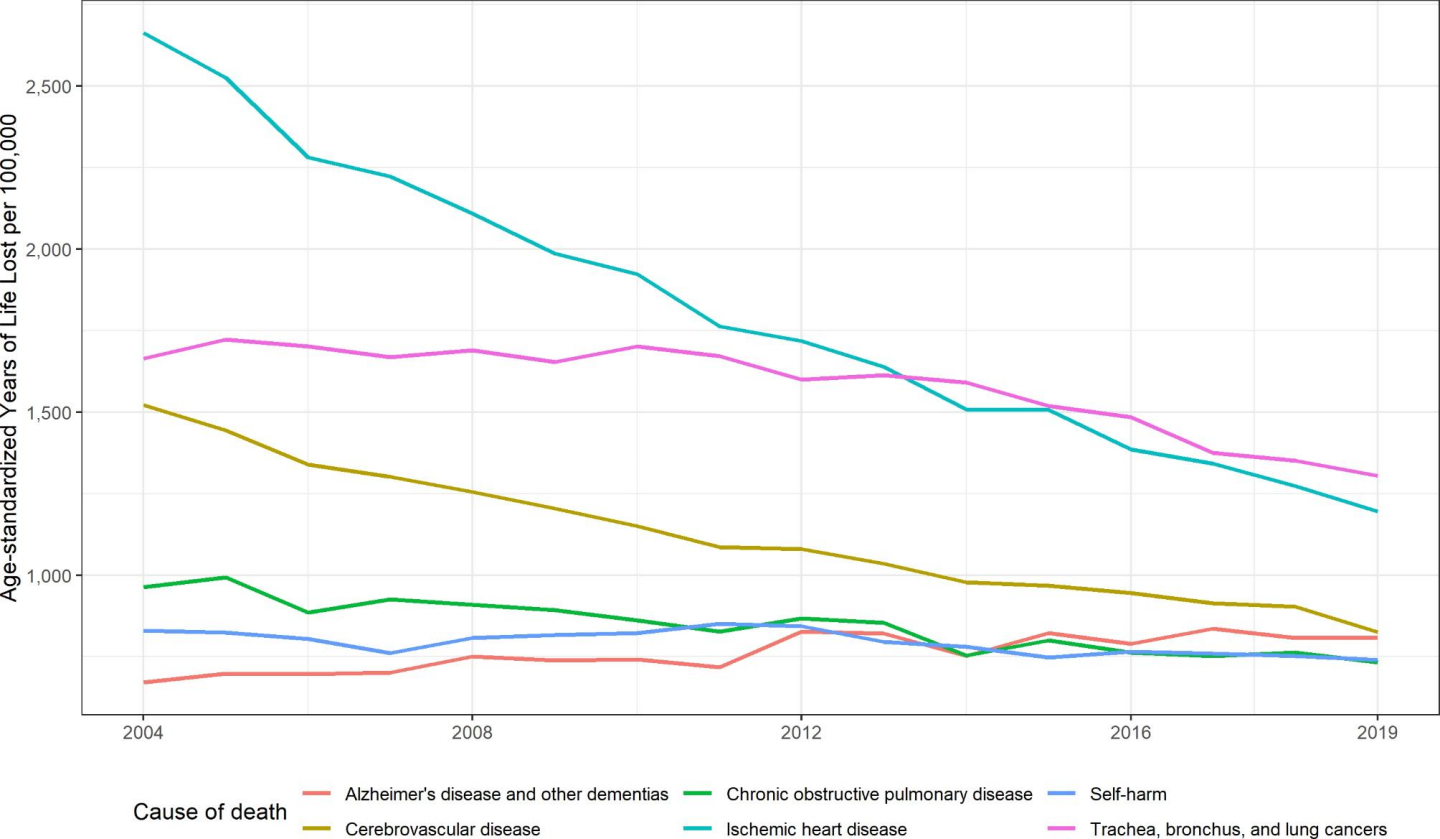
Age-standardized years of life lost rates (per 100,000 inhabitants) for the six leading causes of SEYLL in 2019, GBD level 3, both sexes, 2004–2019, Belgium (Reference: 2013 European Standard Population)

Figure 5 shows the ASYRs for the six leading causes of SEYLL for the total Belgian population between 2004 and 2019. Ischemic heart disease was no longer the dominant cause of premature death due to a major decline of 55% from 2,664 to 1,196 SEYLL per 100,000 inhabitants. Lung cancer was the main cause of premature death in 2019, even though ASYRs declined by 22% from 1,664 to 1,306 per 100,000. The ASYRs for cerebrovascular diseases also fell significantly from 1,522 to 826 per 100,000 (-46%). Alzheimer’s disease and other dementias was the 8th leading cause of premature death in 2004 and is almost on par with cerebrovascular diseases due to an increase from 671 to 809 per 100,000 (+ 21%). ASYRs for chronic obstructive pulmonary disease declined by 24% from 963 to 733 per 100 000. ASYRs for self-harm showed a modest decline of 11% with 829 to 741 per 100,000.

When examining the leading causes of death by ASYRS by sex (not shown), the results showed some differences in the ranking from the mid-2010s onward. Between 2004 and 2013, ischemic heart disease was the main cause of premature deaths among both males and females, although a strong decline in male and female ASYRs were already apparent. Since 2013, lung cancer was consistently the main cause of premature death among males. For females, results from the mid-2010s showed ischemic heart disease and breast cancer were the leading causes of premature mortality. In 2019, lung cancer was the leading cause of premature death among females for the first time. Female ASYRs for lung cancer increased by 28% from 665 to 2004 to 855 per 100,000 in 2019. A marked rise was also observed for Alzheimer’s disease and other dementias among females: ASYRs increased from 671 to 810 per 100,000 (+ 21%), making it the second leading cause of premature mortality among females in 2019. The top 5 premature mortality causes for females further included breast cancer, cerebrovascular disease, and ischemic heart disease. For males, ischemic heart diseases, self-harm, chronic obstructive pulmonary disease, and cerebrovascular disease completed the top 5 in 2019.

**Fig. 6:**
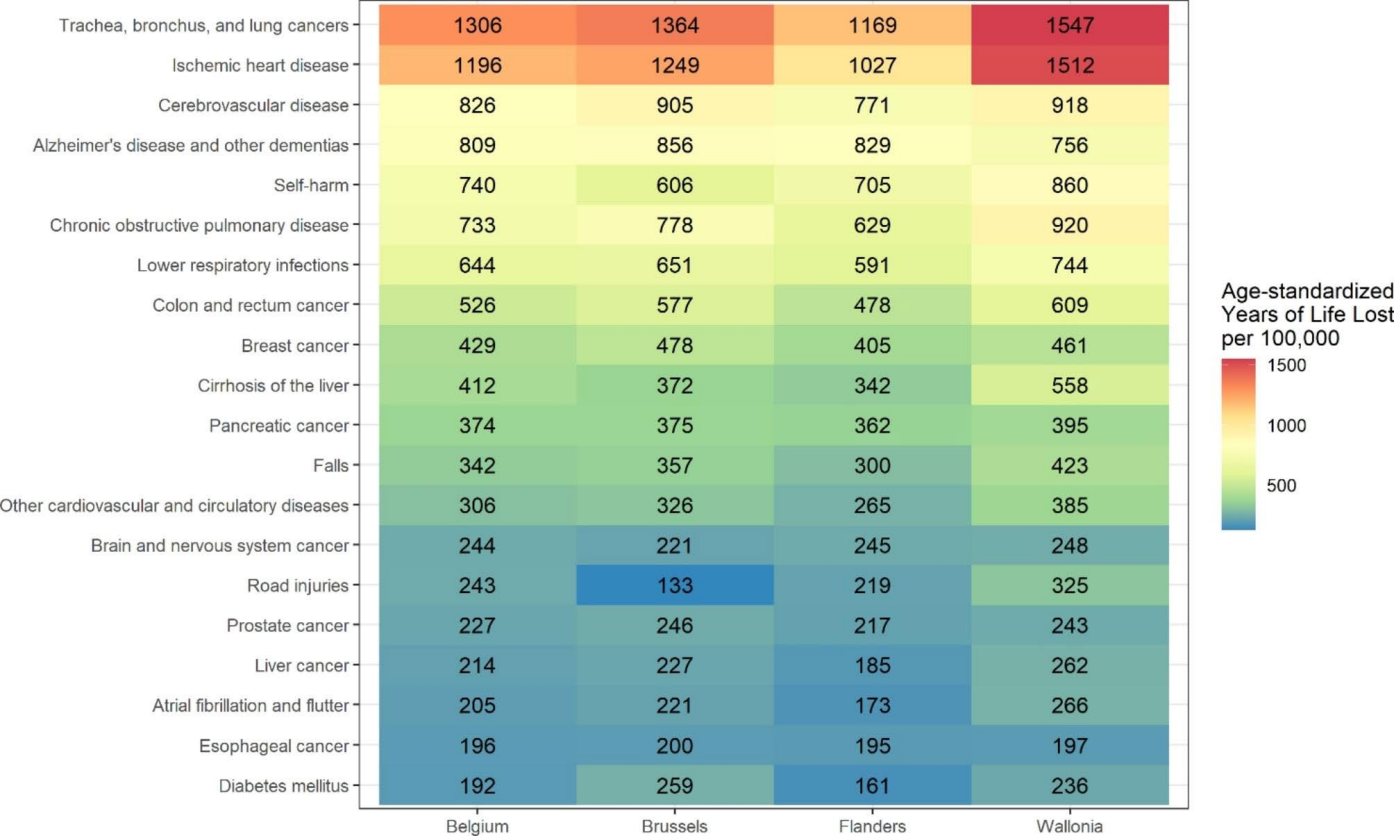
Leading causes of premature death ranked by age-standardized standard expected years of life lost (ASYRs) (per 100,000 inhabitants) for Belgium and the Belgian regions, both sexes, ranked by Belgian ASYRs, GBD level 3, 2019, Reference: 2013 European Standard Population

Figure 6 presents the ASYRs by region. Higher ASYRs were found for most causes in the Walloon Region. The regional patterns of leading causes of premature death were in line with the pattern found for Belgium. One notable exception was chronic obstructive pulmonary disease, which was ranked third in the Walloon Region compared with the sixth place in the Belgian ranking. The Brussels Capital Region had the highest ASYRs for Alzheimer’s disease and other dementias (856 per 100,000) and prostate cancer (246 per 100,000). Road injuries and self-harm were considerably lower in the Brussels Capital Region.

## Discussion

Ill-defined deaths (IDDs) provide a challenge for estimating BoD at national and international levels [12]. We found that, in Belgium, as much as 34% of all deaths are to be considered as IDDs, according to GBD definitions. A potential explanation for this finding can be found in Belgium’s epidemiological profile, with an overall high prevalence of cardiovascular diseases, and an ageing population. Belgium’s ageing population is reflected in the mortality statistics, with over two-thirds of all deaths occurring at advanced ages. According to Meslé and Vallin, ICD coding is less straightforward for deaths at old age compared to younger ages [25]. Deaths at old ages are often the result of a general deterioration of health or a complex multifactorial chain of events in which no specific cause is decisive. Furthermore, medical investigations are limited in the elderly and therefore precise diagnoses are often lacking. Also, coders do not contact the certifying physician for IDDs above 80 years of age. Previous international redistribution studies also indicate high shares of redistributed deaths to causes of death related to old age [9, 28]. In light of the ageing societies in most world regions, the importance of redistribution techniques in mortality research is expected to increase even further.

We developed and adopted a new method for redistributing these IDDs making optimal use of the available multiple causes of death data. This method consists of four steps, referred to as ICD-based redistribution, package redistribution, internal distribution, and all-cause redistribution. This approach is tailored to the availability and quality of the Belgian multiple causes of death data, and may therefore not be directly applicable in other countries. In Germany, for instance, there is no national database that includes multiple causes of death data, which implies that our approach would not be applicable [9].

Our four-step procedure redistributed for 2019 the total 34% of IDDs, with 14% using a priori defined target codes, 11% using *a posteriori* defined target codes for newly developed packages, 1% using internal redistribution, and 8% across all target codes. The most common IDDs were I50.9 (Heart failure, unspecified), followed by J18.9 (Pneumonia, unspecified organism), and R99 (Ill-defined and unknown cause of mortality), corresponding well to international findings [4]. Overall, most IDDs were redistributed to lower respiratory infections, cerebrovascular disease, and ischemic heart disease. The high share of redistributed deaths for lower respiratory infections and cerebrovascular disease can be explained by the IDD definitions used in the GBD study. J18.9 (“Pneumonia, unspecified organism”) and I64 (“Stroke, not specified as haemorrhage or infarction”) are defined as IDDs at level 4 of the GBD cause list only, while being specific for the level 3 GBD causes of “lower respiratory infections” and “cerebrovascular disease”, respectively. This implies that deaths with J18.9 or I64 as underlying cause of death will be considered IDDs, but will be fully redistributed to their corresponding level 3 causes. The high share of redistributed deaths for ischemic heart disease, on the other hand, is mainly due to the high number of IDDs referring to heart failure, which is common and relatively frequently redistributed to ischemic heart disease.

Using the redistributed data, the Belgian fatal burden disease between 2004 and 2019 has been investigated. Overall, ischemic heart disease, cerebrovascular disease and lung cancer had the largest contribution to the Belgian mortality burden. Although these diseases were consistently ranked in the leading 10 causes of death in previous examinations [29–31], the redistribution method now estimates their full impact on mortality in Belgium. Mortality rates for most leading causes of death decreased over time. In contrast, Alzheimer’s disease and other dementias ASMRs increased for both males and females during the observation period. For females, results also show a marked increase in lung cancer over time.

When comparing our results with the GBD estimates for Belgium, we find similar redistributed shares of deaths per specific cause, but also some remarkable differences [32]. For example, the presented method estimates that 9% of all deaths in 2019 are due to ischemic heart diseases, whereas GBD estimates are considerably higher with 13%. It seems that the presented method is more appropriate in estimating the proportion of deaths due to Alzheimer’s disease and other dementias, as GBD results for these diseases show very large confidence intervals for the 2019 estimate of 5.8% (1.5–15.2%), compared with our estimate of 8.9% (8.8–9.0%). With regard to the SEYLL estimates, the GBD redistribution method led to higher estimates of the premature mortality burden in Belgium for ischemic heart disease (1755 vs. 1218 per 100 000), cerebrovascular disease (995 vs. 851 per 100 000), and lung cancer (1400 vs. 1292 per 100 000); and underestimate the burden for Alzheimer’s disease and other dementias (534 vs. 859 per 100 000). However, since full details of the GBD distribution methods by country are lacking, it is not possible to fully investigate and understand these differences.

The results further confirm previous findings related to sex differences in the (premature) mortality pattern in Belgium [31]. Despite converging trends for male and female rates, males experience more (premature) deaths compared with females. In 2019, the main cause of death for males was ischemic heart disease and for females Alzheimer’s disease and other dementias. The main cause of premature mortality in 2019 was lung cancer, for both males and females. Differential (premature) mortality patterns were also found by region with the highest rates observed for the Walloon region and the lowest for the Flemish region. The Brussels Capital Region held an intermediate position. Despite some differences in the ranking, mortality in all the regions is impacted mostly by ischemic heart disease, cerebrovascular disease, lung cancer and Alzheimer’s disease and other dementias. A similar pattern is found for premature mortality, apart from a larger contribution of chronic pulmonary diseases to the premature mortality burden in the Walloon Region. Understanding the impact of these mostly preventable diseases can be instrumental for the further development of targeted public health strategies.

This study entails several limitations. First, the methodology was developed in the Belgian context and the further applicability in other (sub)national contexts largely depends on the available data. However, research efforts are currently under way to adapt this methodology to other European countries, within the context of the European Burden of Disease Network [33]. Second, the study follows the official definition of mortality as defined by Statistics Belgium. The final database combines mortality of Belgian residents and deaths of Belgian nationals occurring abroad since 2010. Previously, only deaths occurring among Belgian residents were included. The combination of a territorial definition (place of death) and a residence-based (National Register) definition has a marginal impact on the redistribution method. Second, the choice of a life table has been shown to affect the estimates [12]. We used an aspirational life tables developed by GBD to facilitate the comparison with other country-specific results. Third, the probabilistic nature of the redistribution method requires increased computational capacity. To measure the uncertainty, the redistributions are simulated in 100 iterations. Sensitivity analyses were performed using 200 iterations, providing very similar mean number of deaths per specific cause (difference < 0.001), but larger confidence intervals (difference ~ 2%).

The strength of the study lies in the development of a transparent and adaptable redistribution method. Using this method, this study provides the first quantification of the Belgian BoD building on all available mortality data. The estimates and their confidence intervals are furthermore made freely available in an online tool to further improve the exploration of the temporal and geographical patterns in (premature) mortality. Future developments of this approach will include considering temporal patterns in IDDs, differences in socio-economic positions, estimating risk factor attributions, making forecasts, and combining these estimates with estimates of Years Lived in Disability, to compute Disability-Adjusted Life Years.

## Conclusion

We estimated, for the first time, the fatal burden of disease in Belgium, based on a redistribution of deaths with ill-defined underlying cause of death. Our approach consists of four consecutive steps and takes optimal advantage of the available multiple causes of death data. Ischemic heart disease, Alzheimer’s disease and other dementias, and cerebrovascular diseases are the leading causes of death, although the importance of cardiovascular diseases is decreasing while that of Alzheimer’s disease and other dementias is increasing. Leading causes of premature mortality are lung cancer, ischemic heart disease, and Alzheimer’s disease and other dementias. Our results also confirm persisting mortality differences between sexes and Belgian regions. Annual updates of our estimates will provide a powerful monitoring tool, which can be further expanded in scope by integrating social inequalities and risk factor attributions.

### Electronic supplementary material

Below is the link to the electronic supplementary material.

Supplementary Material 1

## Data Availability

All estimates are available in an online tool https://burden.sciensano.be/shiny/mortality2019/. The underlying microdata are available from Statistics Belgium by following the procedures described on https://statbel.fgov.be/en/about-statbel/what-we-do/microdata-research.
